# Definition of Normal Vertebral Morphometry Using NHANES‐II Radiographs

**DOI:** 10.1002/jbm4.10677

**Published:** 2022-09-27

**Authors:** John A. Hipp, Trevor F. Grieco, Patrick Newman, Charles A. Reitman

**Affiliations:** ^1^ Medical Metrics, Imaging Core Laboratory Houston TX USA; ^2^ Orthopaedics and Physical Medicine Medical University of South Carolina Charleston SC USA

**Keywords:** CERVICAL, LUMBAR, NORMAL, VERTEBRAL MORPHOMETRY

## Abstract

A robust definition of normal vertebral morphometry is required to confidently identify abnormalities such as fractures. The Second National Health and Nutrition Examination Survey (NHANES‐II) collected a nationwide probability sample to document the health status of the United States. Over 10,000 lateral cervical spine and 7,000 lateral lumbar spine X‐rays were collected. Demographic, anthropometric, health, and medical history data were also collected. The coordinates of the vertebral body corners were obtained for each lumbar and cervical vertebra using previously validated, automated technology consisting of a pipeline of neural networks and coded logic. These landmarks were used to calculate six vertebral body morphometry metrics. Descriptive statistics were generated and used to identify and trim outliers from the data. Descriptive statistics were tabulated using the trimmed data for use in quantifying deviation from average for each metric. The dependency of these metrics on sex, age, race, nation of origin, height, weight, and body mass index (BMI) was also assessed. There was low variation in vertebral morphometry after accounting for vertebrae (eg, L1, L2), and the *R* ^2^ was high for ANOVAs. Excluding outliers, age, sex, race, nation of origin, height, weight, and BMI were statistically significant for most of the variables, though the F‐statistic was very small compared to that for vertebral level. Excluding all variables except vertebra changed the ANOVA *R* ^2^ very little. Reference data were generated that could be used to produce standardized metrics in units of SD from mean. This allows for easy identification of abnormalities resulting from vertebral fractures, atypical vertebral body morphometries, and other congenital or degenerative conditions. Standardized metrics also remove the effect of vertebral level, facilitating easy interpretation and enabling data for all vertebrae to be pooled in research studies. © 2022 The Authors. *JBMR Plus* published by Wiley Periodicals LLC on behalf of American Society for Bone and Mineral Research.

## Graphical abstract

Reference data are needed to define normal lumbar and cervical vertebral morphometry. Reference data were developed based on an anlysis of over 17,000 lumbar and cervical x‐rays collected in a national Health survey. Morphometric data were calculated from anatomic landmarks produced by neural networks and coded logic. Covariates and error sources were explored.

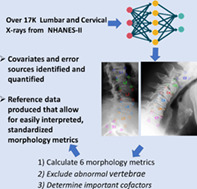

## Introduction

Although vertebral fractures can be asymptomatic, such fractures can be a source of symptoms. One of the most important predictors of subsequent fractures is the number of prior fractures.^(^ ^1^, ^2^, ^3^ ^)^ Vertebral fractures are therefore diagnostically important. Fractures can be difficult to diagnose and are missed in clinical practice.^(^ ^4^, ^5^, ^6^ ^)^ Ferrar et al. succinctly identified the challenge of fracture detection: “The identification of vertebral fractures is problematic because^(^ ^1^ ^)^ ‘normal’ radiological appearances in the spine vary greatly both among and within individuals^(^ ^2^ ^)^; ‘normal’ vertebrae may exhibit misleading radiological appearances due to radiographic projection error; and^(^ ^3^ ^)^ ‘abnormal’ appearances due to non‐fracture deformities and normal variants are common, but can be difficult to differentiate from true vertebral fracture.”^(^ ^7^ ^)^

A typical definition of a fracture is “20% or greater loss in expected vertebral body height.” This definition requires definition of “expected” vertebral body height, and that in turn requires accurate measurements of vertebral body shape in a large and representative population. Validation of the clinically meaningful threshold level for change in vertebral dimensions also requires a reliable reference standard. Inaccuracies in measuring shape and the challenge of finding a suitable reference standard are well documented.^(^ ^7^, ^8^, ^9^, ^10^, ^11^, ^12^, ^13^, ^14^, ^15^, ^16^, ^17^, ^18^, ^19^, ^20^, ^21^, ^22^, ^23^, ^24^, ^25^, ^26^, ^27^, ^28^, ^29^, ^30^, ^31^, ^32^, ^33^, ^34^, ^35^, ^36^, ^37^, ^38^, ^39^, ^40^, ^41^, ^42^, ^43^ ^)^ One option is to use the patient's own adjacent vertebral bodies as a reference, but there is no definitive method for assuring that the adjacent levels are normal or reference data to know how much variability between levels is normal. Normative vertebral body shape metrics that represent a large proportion of the population and account for age, sex, and other confounding factors have not been previously published. Robust reference data and an understanding of the important confounding factors would facilitate more accurate and reproducible diagnosis of the presence and severity of vertebral body fractures.

Between 1976 and 1980, the Second National Health and Nutrition Examination Survey (NHANES‐II) was conducted.^1^ This was a nationwide probability sample to document the health status of the United States. Justified by the prevalence and societal impact of neck and back pain, approximately 10,000 lateral cervical spine and 7,000 lateral lumbar spine X‐rays were collected as part of this survey. Demographic, anthropometric, health, and medical history data were also collected. This resource can be used for establishing normative reference data that can subsequently be used to diagnose abnormalities such as fractures, vertebral deformities, and congenital abnormalities. These data can also be used to determine whether the sex, age, race, height, weight, or body mass index (BMI) of an individual is needed to predict normal vertebral morphometry.

## Methods

**Fig. 1:**
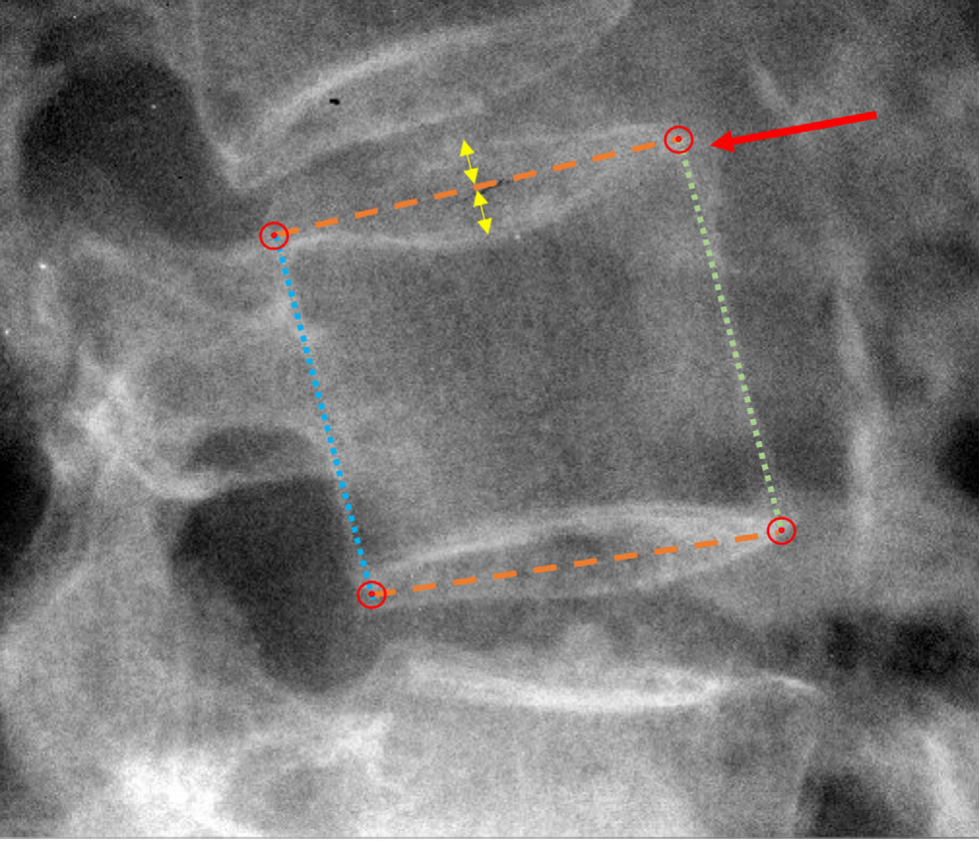
Details of anatomic landmark placement. Dashed lines show assumed midsagittal plane of superior and inferior endplates, identified as bisecting the radiographic shadows of the left and right sides of the endplates (yellow arrows). The red circles show the four landmarks used to measure vertebral body morphometry. The red arrow points to an anterior osteophyte that is ignored. The dotted lines show the anterior and posterior vertebral body heights.

The NHANES‐II images and data were obtained through public access.^2^ All images were anonymized and identified only by a study ID. A previously validated series of neural networks and coded logic (Spine Camp, Medical Metrics, Inc., Houston, TX) were used to obtain four landmarks for each vertebral body from C2 to C7 and L1 to S1 (Fig. 1). Although the NHANES‐II lumbar X‐rays almost always included up to T10 in the field of view, the neural networks were only trained to recognize L1 to S1. The cervical spine neural networks were only trained to find C2 to C7. The neural networks include quality control checks to assure the X‐ray was a lateral lumbar view and manipulate the image if needed so upper vertebrae are toward the top of the X‐ray, with spinous processes toward the left side and with bone being whiter than air. Neural networks and coded logic were then used to segment the bone, find individual vertebrae, label the vertebrae, find the four corners, and finally refrain from reporting landmark coordinates when the confidence of the networks was too low. All the neural networks were trained with over 50,000 lateral X‐rays where analysts had previously digitized standardized landmarks. The analysts used Quantitative Motion Analysis software (QMA, Medical Metrics, Inc., Houston, TX) to place the landmarks. The QMA software was previously validated.^(^ ^44^, ^45^, ^46^, ^47^, ^48^, ^49^ ^)^ NHANES‐II images were not used in training the neural networks.

Details of landmark placement are provided in what follows since, as Keynan et al. noted, “There is a lack of standardization in the literature regarding choice and technique for the measurement of these parameters.”^(^ ^38^ ^)^ The standardized landmark placement was focused on the midsagittal plane of each vertebral body (Fig. 1), excluding residual uncinate processes, and placing landmarks that identify the corners of the vertebral bodies prior to any osteophyte formation.^(^ ^50^, ^51^ ^)^ Landmark placement was similar to that illustrated in Fig. 3 of Genant et al.^(^ ^52^ ^)^ The posterior superior landmarks were placed so as to represent the endplate as it would appear on a midsagittal slice of a CT exam, rather than up at the top of the posterior ridges on the upper endplate, like the logic described in Keynan et al.^(^ ^38^ ^)^ When the X‐ray beam path through a vertebra is not perpendicular to the midsagittal plane of the vertebra, the left and right sides of the vertebral endplates or the left and right aspects of the posterior vertebral body may be seen on the X‐ray.^(^ ^17^, ^21^ ^)^ It is understood that radiographic shadows, which might be interpreted as the left and right rims of the endplates, might not exactly represent the endplate rims,^(^ ^17^ ^)^ but it is assumed that the line bisecting these shadows is the best available radiographic approximation of the midsagittal plane. Therefore, the midsagittal plane is assumed to be midway between the radiographic shadows of the left and right sides of endplate and midway between the radiographic shadows of the left and right aspects of the posterior wall. Multiple examples of landmark placement are provided online at https://www.dropbox.com/sh/qzrocrh86goxarx/AAADRXs5HoEjVdRcGIwA4beIa?dl=0

to help readers appreciate the nuances of how landmarks were placed. Vertebral morphometry calculated from landmarks is dependent on details of landmark placement, and it is therefore important to appreciate landmark placement details. The examples provided include vertebrae with abnormal morphometry (as defined by the metrics presented in this paper). The metrics for each online example are also provided to help readers visualize abnormal morphometry.

The neural networks and coded logic produced landmark coordinates for the vertebral bodies from L1 to S1 in lumbar spine radiographs and C2 to C7 in cervical spine radiographs. An anomaly detection algorithm was then run on those data to identify anomalies due to very poor image quality, severe image artifacts, and very unusual anatomy. Those anomalies were removed from subsequent data analysis.

The coordinates of the four vertebral body corners were used to calculate six vertebral body morphometry metrics that were used in prior studies:

1. anterior/posterior vertebral body height ratio (VBHR),
2. superior/inferior endplate width ratio (EPWR),
3. forward/backward diagonal ratio (FBDR),
4. height/width ratio (HWR),
5. angle between superior and inferior endplates (EPA),
6. angle between posterior wall and superior endplate (PSA).

The average of the anterior and posterior heights and the average of the superior and inferior endplate widths were used to calculate the HWR. The metrics are illustrated in Appendix S1: Fig. 1.

The NHANES‐II lumbar radiographs were obtained with the subject in the lateral recumbent position with body flexion.^(^ ^53^ ^)^ Because this is not the typical position used to obtain a radiograph for vertebral fracture detection, global and local spinal lordosis were not calculated. The NHANES‐II cervical spine radiographs were obtained with the subject standing and a 25‐lb weight in each hand to pull down the shoulders and the head and neck flexed forward.^(^ ^53^ ^)^

Principal component analysis was used to determine whether the dimensionality of the data could be reduced (Stata version 15). The dependency of the six vertebral morphometry metrics on sex, age, race, nation of origin, height, weight, and BMI was assessed using ANOVA (Stata version 15). In the NHANES‐II study, race was recorded as white, black, or other. Nation of origin was one of 13 categories.

Descriptive statistics were tabulated and used to identify and trim outliers in the data that might have compromised the definition of “normal” vertebral morphometry.^(^ ^54^, ^55^, ^56^, ^57^ ^)^ The data were trimmed by excluding all vertebrae where any of the six metrics was more than 2 SD above or below the mean, and descriptive statistics were tabulated for use in quantifying deviation from average for each metric. Outliers more than 3 SD above or below the mean were inspected to identify typical explanations for each type of outlier. Correlations between the six morphometry metrics were also explored. Finally, means and SDs were tabulated for each metric, for each vertebra from L1 to S1, and from C2 to C7. Those means and SDs were then used to calculate standardized metrics (SDs from mean) for the entire data sets. All statistical analysis was completed using Stata version 15 (College Station, TX, USA).

The NHANES‐II study also includes a response to the following question: “Have you ever had pain in your back on most days for at least 2 weeks?” The response to the question was analyzed for associations with the six morphometry metrics. A reproducibility study was also performed and is described in Appendix S2. An assessment of errors that can occur in the morphometry metrics due to variability in radiographic projection is described in Appendix S3.

## Results

### Summary of data analyzed

**Fig. 2:**
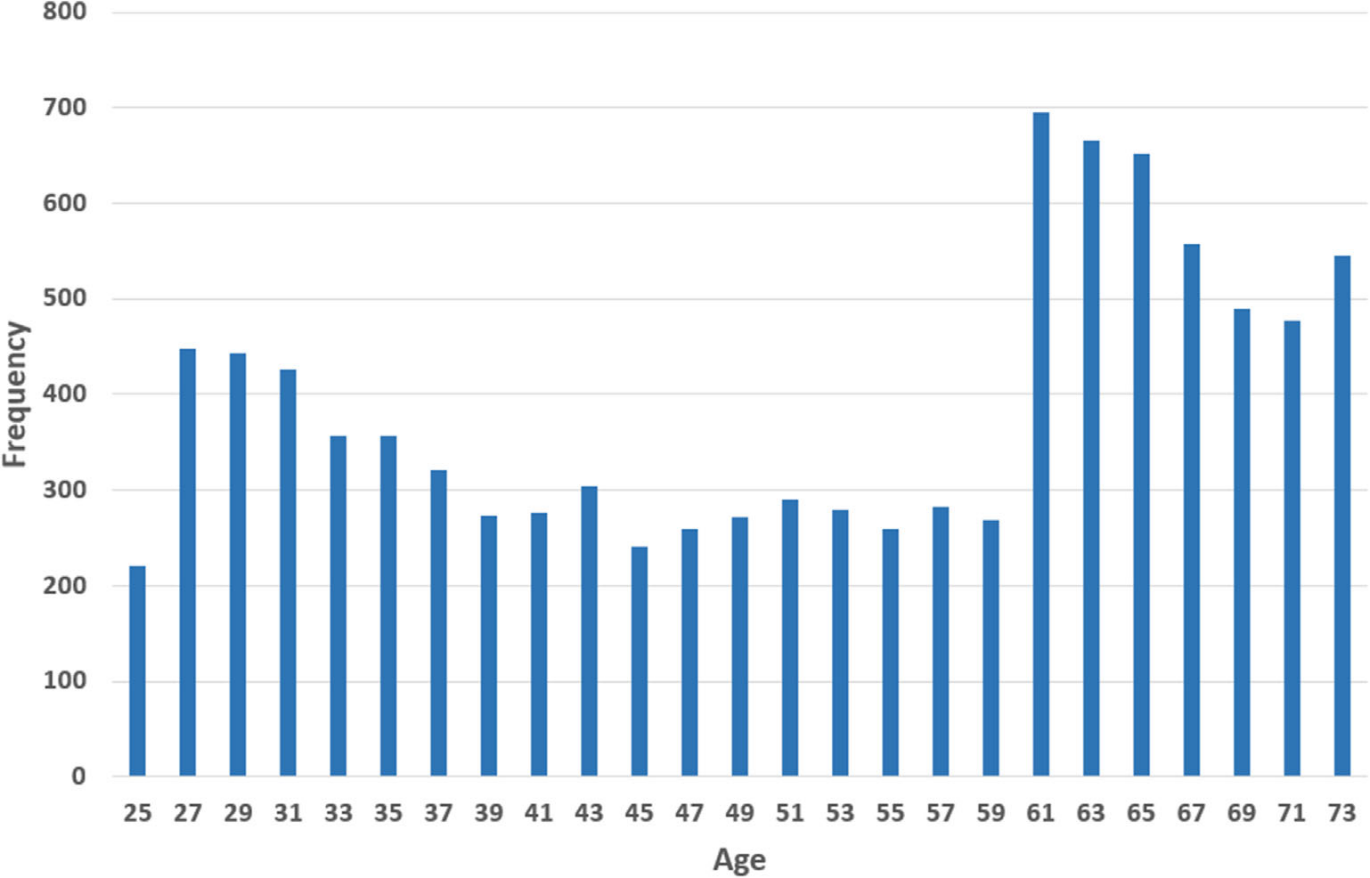
Distribution of ages in Second National Health and Nutrition Examination Survey study.

Computer‐generated lumbar landmarks were obtained for 42,980 vertebrae from 7,364 of 7,423 total lumbar radiographs and 54,093 vertebrae from 9,662 out of 9,669 cervical radiographs for subjects 25–74 years old. Fifty lumbar X‐rays were not analyzed due to an error in file transfer, and seven cervical and nine lumbar X‐rays were nonanalyzable. Landmark generation for all lumbar and cervical radiographs took 22 hours on a dual computer processing unit (CPU), four graphics processing unit (GPU) (Nvidia T4) production server. The age range was 25–74 years, and the demographic and body size data are summarized in Table 1. The ages of the subjects (using cervical data) were also biased toward older ages (Fig. 2).

**Table 1:** Sample size, average age [SD], and average BMI (kg/m^2^) [SD]

Sex	Lumbar			Cervical		
	N	Age	BMI	N	Age	BMI
Male	4,553	50.9 [15.3]	25.5 [4.0]	4,610	50.8 [15.3]	25.5 [4.0]
Female	2,809	63.3 [6.4]	26.4 [5.5]	5,050	51.4 [15.2]	25.8 [5.8]

Age and BMI were missing for two subjects.

Abbreviation: BMI, body mass index.

Note that females are underrepresented in the lumbar data. By the design of the NHANES‐II study, no lumbar X‐rays were taken for pregnant women or women under 50.^(^ ^58^ ^)^ An additional limitation relates to the data on race and nation of origin recorded in the NHANES‐II data. Based on the cervical data, 86.9% of NHANES‐II subjects were “white,” 11.2% were “black,” and the rest were “other.” A more uniform representation of races would likely be needed to fully understand the importance of race. The same is true for the “nation of origin” data in NHANES‐II. Note that the text in quotes is as it appears in the NHANES‐II documentation: 74.1% of subjects are classified as “OTHER EUROPE SUCH AS GERMAN, FRENCH, ENGLISH, IRISH,” 10.8% as “BLACK, NEGRO OR AFRO‐AMERICAN,” and the rest distributed in one of 11 other classifications.

Principal component analysis (PCA) of the lumbar data documented that five primary components explained 99.8% of the variance in the morphometry data, and PCA of the cervical data showed that six components explained 99.7% of the variance. Since PCA does not appreciably reduce the dimensionality of the data, they were not investigated further.

### Descriptive statistics for normative reference

**Fig. 3:**
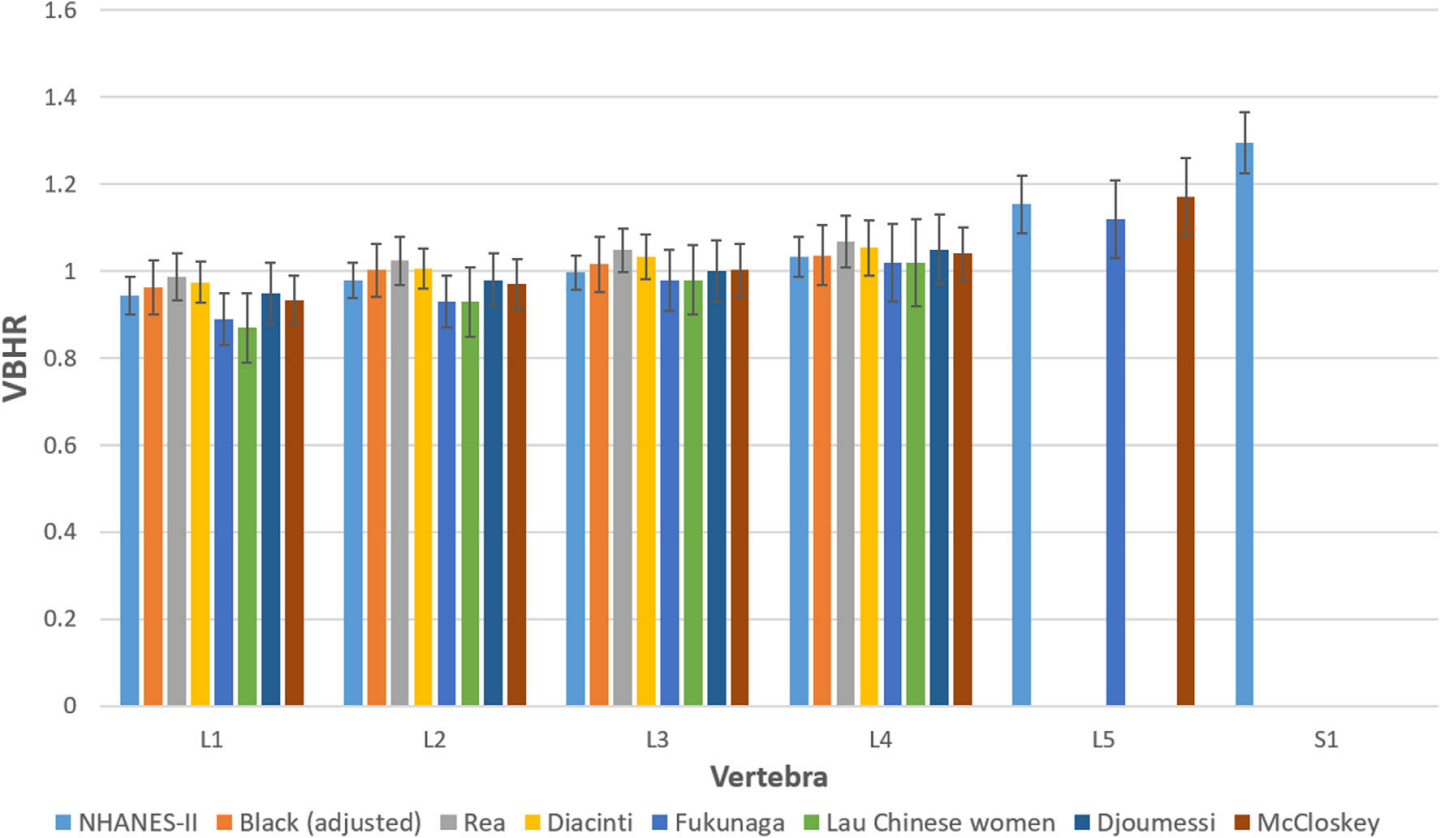
Comparison of previously published vertebral body height ratio data with data obtained from Second National Health and Nutrition Examination Survey.^(^ ^54^, ^55^, ^56^, ^57^, ^59^, ^60^, ^61^ ^)^

Descriptive statistics for the morphometric parameters are provided in Appendix S1: Tables 1 and 2, after trimming the data. Mean values changed very little for the morphometry metrics after trimming the data, suggesting that normal morphometry is dominant in the data. SDs were reduced after trimming. These observations are the same as reported in a similar study.^(^ ^56^ ^)^ Data with a Gaussian distribution have a skewness of 0 and kurtosis of 3. Trimming the data substantially reduced the skewness from an average of 0.27 to 0.05 and substantially narrowed the tails of the distribution from an average kurtosis of 4.1 to 2.5. This shows that the distribution of data was more Gaussian after trimming. There was remarkably small variation in vertebral morphometry after accounting for vertebrae (eg, L1, L2). The PSA, FBDR, and EPWR generally had the lowest coefficients of variation (Appendix S1: Tables 3 and 4). Figure 3 provides a comparison of VBHR data in Appendix S1: Table 4 with some previously reported morphometry metrics.^(^ ^54^, ^55^, ^56^, ^57^, ^59^, ^60^, ^61^ ^)^

### Effect of covariates

ANOVA (after trimming the data) revealed that age, sex, race, and nation of origin were statistically significant for most of the variables (Appendix S1: Tables 5 and 6), and the *R* ^2^ was high for most variables. However, the F‐statistic was generally small for all variables except vertebral level. Excluding all variables except vertebral level in the ANOVAs changed the *R* ^2^ very little (eg, for the lumbar data, VBHR *R* ^2^ went from 0.85 to 0.84 and HWR *R* ^2^ went from 0.84 to 0.80). This shows that age, sex, race, and body size are statistically significant but contribute relatively little to the variability in the morphometry metrics. Therefore, it may be reasonable to pool data across sex, race, and nation of origin, at least for the NHANES‐II data.

The six morphometric variables were significantly correlated with each other (Appendix S1: Tables 7 and 8). EPA and VBHR were almost perfectly correlated, suggesting that only one of these two variables needs to be analyzed. Appendix S1: Tables 9 and 10 provide reference data for the difference between levels in the morphometry metrics.

### Abnormalities in NHANES data

Mean and SD data from Appendix S1: Tables 1 and 2 were used to calculate the vertebra‐specific Z‐score for each metric according to the following equation:

$$Z=\frac{x-\mu }{\sigma }$$

where x is the metric for a vertebra, μ is the vertebra‐specific mean for the corresponding metric, and σ is the vertebra‐specific SD for the corresponding metric. Based on these Z‐scores, 28,813 of 42,980 lumbar vertebrae (67%) were within ±2 SD of average for all six of the metrics. These numbers are less than obtained after trimming since the SDs in Appendix S1: Tables 1 and 2 are smaller than originally used to identify outliers and are therefore more sensitive to abnormalities. Similarly, 36,869 of 54,093 cervical vertebrae (68%) were within ±2 SD of average for all six of the metrics.

Box and whisker plots illustrate the magnitude of outliers that exist in the NHANES data (Appendix S1: Figs 2 and 3). Note that the medians are near zero and generally centered within the interquartile range and the whiskers are symmetric. Positive VBHR outliers represent posterior wedge‐shaped vertebral bodies, whereas negative VBHR outliers represent anterior wedge‐shaped vertebrae. The wedging may be from fractures, remodeling, or congenital conditions, and identifying a specific cause would require more than a single radiograph. Positive EPWR outliers represent vertebrae that are unusually wide superiorly versus inferiorly, and negative outliers are the opposite. Positive and negative FBDR outliers generally appear to be best explained by remodeling, perhaps after a fracture that caused splaying of the fracture fragments. Positive HWR outliers appear as unusually tall vertebrae, whereas negative HWR outliers generally appear to be best explained by a crush fracture. Positive EPA outliers appear as anteriorly wedge‐shaped vertebrae, whereas negative outliers appear as posteriorly wedge‐shaped vertebrae. Positive PSA outliers generally appear as posterior wedge‐shaped vertebrae, and negative outliers generally appear as anteriorly wedge‐shaped vertebrae. It is not possible to definitively identify the cause of each abnormality from a single radiograph. Examples of each of high positive and low negative outliers, for each of the six metrics, including landmarks, can be viewed online: https://www.dropbox.com/sh/qzrocrh86goxarx/AAADRXs5HoEjVdRcGIwA4beIa?dl=0

### Additional observations

Based on logistic regression, the subject response to the question: “Have you ever had pain in your back on most days for at least 2 weeks” was significantly (*p* < 0.05) associated with age, race, and BMI. Since there is no way to determine which vertebra (if any) might be causing back pain, the sum of abnormalities for each subject (metrics < −2 or > 2 SD from the average based on Appendix S1: Table 1) from L1 to S1 was calculated. This sum of abnormalities for the EPWR and the FBDR was significantly associated with the response to the “ever had back pain” question (*p* < 0.05), although the *R* ^2^ for these regressions was very small (>0.01), indicating a very weak association.

Of the six morphometry metrics, FBDR was the most sensitive and specific metric (area under ROC curve = 0.995) to use for the purpose of using landmarks to classify vertebrae as S1 versus L1–L5. A threshold level of FBDR > 1.0574 misclassified only 278/42,980 vertebrae as S1 versus L1–L5. Including all data for all vertebrae, the proportions of vertebrae with abnormal morphometry (<>2 SD from average based on Appendix S1: Tables 1 and 2) are summarized in Appendix S1: Tables 11 and 12.

## Discussion

Vertebral morphometry was calculated from landmarks marking the four corners of lumbar and cervical vertebrae. These data may help to establish a reference standard for normal vertebral morphometry that can be used to identify abnormal morphometry, such as can occur as a result of a fracture. The landmarks were produced using a proprietary network of neural networks and coded logic. However, the landmarks are intended to be equivalent to landmarks produced for many prior research studies. Artificial intelligence (AI) has been used to produce vertebral landmarks in many prior studies.^(^ ^62^, ^63^, ^64^, ^65^, ^66^, ^67^ ^)^ It is expected that the landmark placement obtained for the NHANES‐II radiographs can be reproduced by other methods, and the NHANES‐II reference data in Appendix S1 will in this way be useful in other studies where landmarks are obtained with alternative methods.

The NHANES‐II morphometry data may help in addressing the challenge of accounting for normal variants when assessing for vertebral fractures.^(^ ^51^, ^68^ ^)^ For example, a somewhat wedge‐shaped L5 may be considered a normal variant in clinical practice. Accurate measurements, along with reliable reference data, will help to minimize uncertainty about whether wedging is within normal limits. The question of how much deviation from normal requires clinical intervention remains a challenge. Standardized metrics along with reliable reference data may facilitate the development of computer‐aided diagnosis technology.^(^ ^69^ ^)^

It would have been valuable to obtain data on thoracic vertebral morphometry. The NHANES‐II X‐rays did capture the whole thoracic spine, and the technology used to obtain landmarks was not trained to place landmarks on thoracic vertebrae, so thoracic vertebral morphometry could not be obtained.

The NHANES‐II vertebral morphometry data were based on analysis of lateral spine X‐rays. Vertebral morphometry is also assessed from DXA images. The extent to which vertebral morphometry data from X‐rays can be applied to DXA scans is poorly understood. This issue has been addressed to an extent,^(^ ^70^, ^71^, ^72^ ^)^ though no definitive guidance is available. The errors due to variability in radiographic projection described in Appendix S3 are not the same as will occur with DXA scanners, where there would be either no magnification or sagittal plane–only magnification, depending on the scanner. Digitally reconstructed pencil beam, fan beam, and narrow‐angle fan beam DXA images could in theory be created from the same CT studies used in Appendix S3 and errors analyzed and compared to X‐ray errors. However, that was outside the scope of the study. Diacinti et al. and Rea et al. have published vertebral morphometry data from DXA scanners.^(^ ^55^, ^57^ ^)^ There are similarities and differences in their reference data for the anterior/posterior vertebral body height ratios for L1 to L4 with VBHR data in Appendix S1. However, further research is needed to determine the applicability of the NHANES‐II data to other than lateral spine X‐rays.

When the x‐ray beam is not coplanar with the endplates and the posterior wall of the vertebra, suboptimal “out‐of‐plane” images of that vertebra occur. This can occur with suboptimal patient positioning or with scoliosis or lateral listhesis. These out‐of‐plane images can result in errors in the vertebral morphometry measurements. These errors are explored in Appendices S2 and S3. These errors can be large when the out‐of‐planeness is pronounced, and caution should be used when interpreting morphometry in these cases.

Considering the correlation between morphometric variables documented in Appendix S1: Tables 7 and 8, VBHR and EPA were almost perfectly correlated, but the other metrics were not. That observation supports the idea that, aside from VBHR and EPA, each of the metrics independently describes some unique aspect of vertebral morphometry. It cannot be determined from the NHANES data alone which of the six metrics will prove most valuable in research and clinical practice. It may be worth exploring the utility of each metric, and combinations of metrics, in future studies where the goal is to diagnose fractures or vertebral deformities.

Multiple prior publications report efforts to establish reference data that can be used to help diagnose vertebral body fractures.^(^ ^7^, ^8^, ^9^, ^10^, ^11^, ^13^, ^14^, ^15^, ^16^, ^18^, ^19^, ^20^, ^21^, ^22^, ^23^, ^24^, ^25^, ^26^, ^27^, ^28^, ^29^, ^30^, ^31^, ^32^, ^33^, ^34^, ^35^, ^36^, ^37^, ^38^, ^39^, ^40^, ^41^, ^42^, ^43^ ^)^ Although multiple assessments may need to be considered, such as a break or discontinuity in endplates or cortices, intervertebral angles, listhesis, and canal occlusion,^(^ ^38^, ^73^ ^)^ compression of the vertebral body height is commonly used to help identify fractures. When severe loss of vertebral body height has occurred, clinicians can unanimously agree that a fracture has occurred, but when the fracture is subtle, reference data may be important. Midvertebral landmarks that could be used to measure midvertebral height were not routinely available in the images used to train the neural networks, so midvertebral body heights could not be obtained automatically. Further research is needed to determine the implications of this limitation, although it may be possible to combine morphometric analysis relative to the NHANES reference data with an automated implementation of the Genant classification of fracture morphology.

Change in vertebral shape is considered one of the best indicators of a fracture.^(^ ^7^ ^)^ The NHANES‐II images provide only one radiograph per individual, so change in shape could be explored. Nevertheless, the low variability of vertebral shape within a large population supports the potential for using a single X‐ray to detect abnormal morphometry with reference to the NHANES‐II data. Reporting metrics as SDs from average may also help with interpreting changes in morphometry. It is important to consider the quality and equivalence of radiographic projections when comparing morphometry across time points since variability in radiographic projection can cause potentially misleading apparent changes in morphometry (Appendices S2 and S3). It is also likely that, with advancements in the application of AI to producing spine metrics, the data in Appendix S1: Tables 1 and 2 may provide valuable quality control data to determine whether the AI predictions are aberrant or if caution is warranted in interpreting the results.

Application of the vertebral morphometry metrics, such as in Appendix S1: Tables 1 and 2, to the challenge of documenting vertebral fractures will require defining a threshold level of these metrics that can be used to document the presence/absence of a fracture. Eastell et al. and McCloskey et al. used a threshold of 3 SDs for the VBHR.^(^ ^59^, ^74^ ^)^ Black et al. used a threshold of 20% difference from normal VBHR.^(^ ^26^ ^)^ Genant et al. used the criterion of a 15% difference compared to the mean VBHR of a normal population.^(^ ^52^ ^)^ The average VBHR in Appendix S1: Table 1 for L1 to L4 is about 1 and 1 SD is about 0.042, so a 15% difference as used by Genant et al. would be equivalent to approximately a VBHR Z‐score of 3.5. A gold‐standard test for a vertebral fracture does not exist, so validating a threshold remains a challenge. In addition, L5 may be a special case if the variability in L5 morphometry recognized in the Genant classification confounds the use of a single threshold for all vertebrae. An alternative approach would be to validate a threshold that predicts a positive response to treatment or that predicts subsequent fracture risk. That approach could potentially be retrospectively tested at low cost using automated measurements of morphometry applied to imaging and data from prior studies that collected appropriate imaging. It may also be effective to incorporate a categorical assessment of cortical disruptions or endplate deformations.^(^ ^69^ ^)^ It is likely that these assessments, which are typically made subjectively, can be automated with neural networks given sufficient labeled images for training.^(^ ^75^, ^76^ ^)^

It is reasonable to hypothesize that differences in morphometry between adjacent levels may help to identify abnormal morphometry. For example, if one level has an anterior wedge fracture, then a significant difference in the VBHR would be expected when compared to the immediately adjacent levels. A problem occurs when adjacent vertebrae are fractured or otherwise abnormal. Comparison to adjacent levels has been a criterion used or proposed by many investigators, so data that could be used to implement adjacent vertebrae comparisons are provided in Appendix S1: Tables 9 and 10. For example, using data in Appendix S1: Table 9, if the VBHR is to be compared between L4 and L5 vertebrae and normal variability is defined as anything within the 95% confidence interval for data from the NHANES‐II study, then the VBHR when comparing L4 to L5 should not vary by more than 0.124 ± 1.96 × 0.059, or 0.007 to 0.24.

Some error in the data from Appendix S1: Tables 1 and 2 may occur as a result of the challenges of vertebral labeling. A systematic approach to definitively labeling vertebrae requires whole spine imaging.^(^ ^77^ ^)^ An X‐ray or CT of the entire spine from occiput to sacrum was not available, so it cannot be certain that vertebrae were optimally labeled if there were other than five lumbar vertebrae transitional vertebrae or other abnormalities.^(^ ^78^, ^79^ ^)^ Spines with other than five lumbar vertebrae are uncommon.^(^ ^80^ ^)^ Transitional vertebrae may be more common,^(^ ^81^ ^)^ but it is unclear whether this would consistently compromise labeling from a lateral spine X‐ray. The assumption is that the vertebrae were correctly labeled in the majority of data used to train the neural networks and, therefore, were correctly labeled for the majority of the NHANES cases. The possibility of mislabeled vertebrae in a minority of cases should not significantly affect the reference data in Appendix S1: Tables 1 and 2. The prevalence of transitional vertebrae reported in the peer‐reviewed literature varies between 4% and 36%.^(^ ^82^, ^83^, ^84^, ^85^, ^86^ ^)^ The prevalence of transitional vertebrae in the NHANES‐II radiographs remains undocumented. It is assumed that the error sources discussed previously were random sources of error and would not appreciably affect the means and SDs in Appendix S1: Tables 1 and 2.

One advantage of the six vertebral morphometry metrics is the lack of dependence on radiographic magnification. All metrics are ratios of single vertebral dimensions or angles between anatomic reference lines and should not vary appreciably with radiographic magnification (assuming sufficient spatial resolution). This is important since radiographic magnification can vary substantially between lumbar X‐rays,^(^ ^87^, ^88^, ^89^ ^)^ and this can result in errors in measurements of vertebral dimensions.^(^ ^27^ ^)^

The NHANES‐II data do not uniformly represent all ages, sexes, races, and nations of origin. In particular, it would be valuable to repeat this analysis on X‐rays that better represent races and nations of origin not well represented in the NHANES‐II data. Djoumessi et al. have reported differences in the VBHR between different nations of origin.^(^ ^56^ ^)^ Other studies showed a dependence of vertebral morphometry on age, gender, race, and other variables, but the relative magnitude of the influence, relative to other covariates, was not described.^(^ ^90^, ^91^, ^92^, ^93^, ^94^ ^)^ That older ages were more common in the NHANES‐II data (Fig. 3) may explain the relatively high rate of vertebrae with at least one abnormal morphometry metric.

## Conclusion

The lateral spine lumbar and cervical radiographs from the NHANES‐II study were used to establish normative vertebral morphometry reference data. These data can be used to help objectively identify vertebral body fractures, with the assumption that fractures will result in significant deviations from large population‐based average vertebral body dimensions. Early diagnosis of fracture allows for early evaluation and prevention, ultimately decreasing deformity as well as risk of future fracture. The data in Appendix S1: Tables 1 and 2 can be used to produce standardized metrics in units of SD from mean. This allows for easy identification of abnormalities resulting from vertebral fractures, atypical vertebral body morphometrics, and other congenital or degenerative conditions and removes the effect of vertebral level, allowing data for all vertebrae to be pooled in research studies. Metrics developed from a large database, such as NHANES‐II, should help with differentiating between fractures and normal variants of shape, as this can be challenging.^(^ ^95^ ^)^ This may be one approach to computer‐aided diagnosis of vertebral fractures that could supplement classification of vertebral shape, endplate fractures, or use of intra‐ or intervertebral height ratios.^(^ ^96^, ^97^ ^)^ Perhaps the greatest challenge is defining the clinically significant fracture that needs to be diagnosed to improve outcomes and lower costs. The deterministic thresholds for standardized metrics that can provide clinically meaningful diagnostic or prognostic information are yet to be determined. These data may also be useful for quality control in technology designed to automatically obtain landmark coordinates and other metrics from medical imaging. This is hopefully a step toward a definitive reference standard that can be universally used in future research. Secondary analysis of existing radiographs may be a promising approach to assessing the efficacy of the reference data.^(^ ^98^ ^)^ With advancements in AI/machine learning, opportunistic analysis of spine X‐rays could be near instant and inexpensive and may help to detect undiagnosed fractures if appropriate reference data are used.^(^ ^99^ ^)^

## Disclosures

John Hipp, Trevor Grieco, and Patrick Newman are employees of Medical Metrics, Inc. No authors have any other relevant conflicts of interest.

## Author Contributions

**Trevor F Grieco:** Data curation; software; writing – review and editing. **Patrick Newman:** Data curation; resources; software. **Charles A Reitman:** Writing – review and editing.

## Ethics Approval

Vertebral morphometry was measured from lateral lumbar and cervical radiographs obtained during the 2nd National Health and Nutrition Survey (1976–1980). That study was designed and conducted to protect the rights of the volunteers, and all subjects in the study signed a consent form, as described in the study documentation: https://wwwn.cdc.gov/nchs/nhanes/nhanes2/manualsandreports.aspx; https://wwwn.cdc.gov/nchs/data/nhanes2/manuals/15a76_79.pdf

### Peer Review

The peer review history for this article is available at https://publons.com/publon/10.1002/jbm4.10677.

## Supporting information


**Appendix S1.** Supporting information.

**Appendix S2.** Supporting information.

**Appendix S3.** Supporting information.

## Data Availability

The morphometric data derived from the landmarks are available upon reasonable request.

## Endnotes

^1^

https://wwwn.cdc.gov/nchs/nhanes/nhanes2

^2^

https://www.cdc.gov/nchs/nhanes/index.htm
