# Supplementary material for: Definition of Normal Vertebral Morphometry Using NHANES‐II Radiographs
Source: JBMR Plus. 2022 Sep 27;6(10):e10677. doi: 10.1002/jbm4.10677 (PMC9549721; doi:10.1002/jbm4.10677)
Supplement: Supplementary file 1 — Appendix S1. Supporting information. [file JBM4-6-e10677-s002.docx]

# Supplemental Appendix 1

Figure 1: Details on the six morphometry metrics calculated from the four anatomic landmarks.


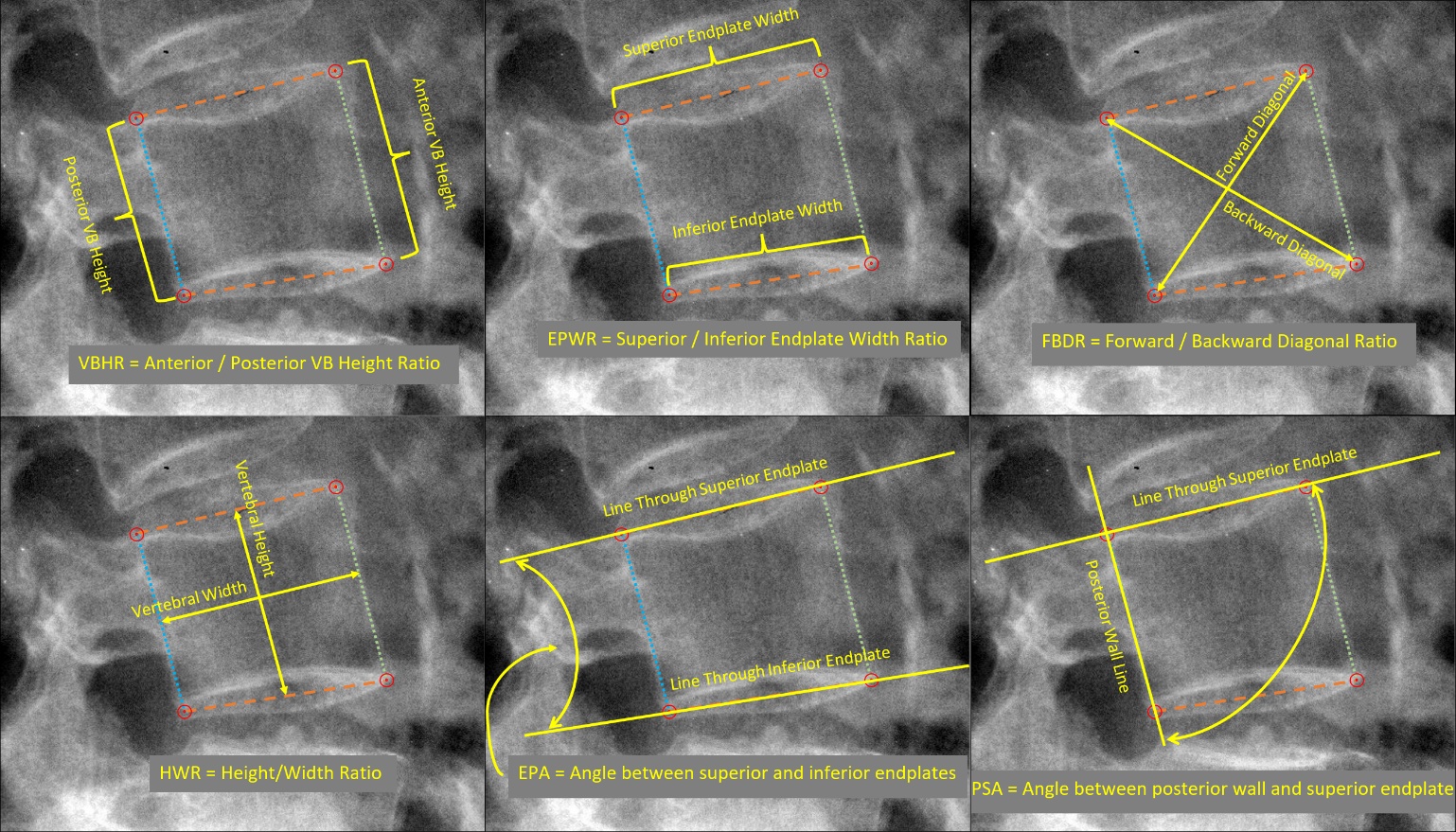


Table 1: Means and [standard deviations] for 35,275 lumbar vertebrae. All vertebrae where any metric was <> 2 SD from the average for all vertebrae in the NHANES-II study were excluded.

| Vert | VBHR | EPWR | FBDR | HWR | EPA | PSA |
| --- | --- | --- | --- | --- | --- | --- |
| L1 | 0.944[0.043] | 0.994[0.017] | 0.994[0.017] | 0.823[0.059] | -2.678[2.035] | 88.499[1.250] |
| L2 | 0.979[0.040] | 0.999[0.018] | 0.973[0.019] | 0.827[0.059] | -1.021[1.907] | 87.919[1.251] |
| L3 | 0.997[0.039] | 1.009[0.017] | 0.960[0.016] | 0.821[0.058] | -0.126[1.825] | 87.222[1.266] |
| L4 | 1.032[0.046] | 1.003[0.019] | 0.938[0.017] | 0.799[0.058] | 1.406[2.049] | 86.753[1.383] |
| L5 | 1.154[0.066] | 1.023[0.025] | 0.930[0.023] | 0.788[0.058] | 6.517[2.655] | 87.671[1.970] |
| S1 | 1.296[0.071] | 1.611[0.154] | 1.268[0.067] | 1.157[0.086] | 11.937[3.456] | 96.564[3.539] |

Table 2: Means and [standard deviations] for 44938 cervical vertebrae. All vertebrae where any metric was <> 2 SD from the average for all vertebrae in the NHANES-II study were excluded.

| Vertebra | VBHR | EPWR | FBDR | HWR | EPA | PSA |
| --- | --- | --- | --- | --- | --- | --- |
| C2 | 1.130[0.042] | 0.790[0.047] | 0.799[0.027] | 1.324[0.083] | 6.389[2.833] | 85.069[2.484] |
| C3 | 0.965[0.047] | 0.935[0.034] | 0.802[0.029] | 0.899[0.076] | -2.810[2.484] | 78.307[2.518] |
| C4 | 0.949[0.045] | 0.945[0.032] | 0.825[0.027] | 0.855[0.069] | -3.322[2.265] | 79.320[2.375] |
| C5 | 0.927[0.043] | 0.929[0.033] | 0.848[0.029] | 0.819[0.070] | -4.354[2.101] | 81.006[2.460] |
| C6 | 0.955[0.044] | 0.952[0.030] | 0.879[0.028] | 0.792[0.072] | -2.521[2.060] | 83.011[2.250] |
| C7 | 0.998[0.035] | 1.009[0.030] | 0.921[0.025] | 0.856[0.064] | -0.108[1.730] | 84.868[1.811] |

Table 3: Coefficients of variation (CV = SD / mean) for 35,275 lumbar vertebrae. Note that the large values for L3 EPA is due to the mean value being very close to zero (ref Table 1) and the CV is difficult to interpret when the denominator is near zero.

| Vertebra | VBHR | EPWR | FBDR | HWR | EPA | PSA | N |
| --- | --- | --- | --- | --- | --- | --- | --- |
| L1 | 0.045 | 0.018 | 0.017 | 0.072 | -0.760 | 0.014 | 5,870 |
| L2 | 0.041 | 0.018 | 0.019 | 0.072 | -1.868 | 0.014 | 5,987 |
| L3 | 0.039 | 0.017 | 0.017 | 0.070 | -14.484 | 0.015 | 5,946 |
| L4 | 0.045 | 0.019 | 0.018 | 0.072 | 1.458 | 0.016 | 5,953 |
| L5 | 0.057 | 0.024 | 0.025 | 0.074 | 0.407 | 0.022 | 5,886 |
| S1 | 0.055 | 0.095 | 0.053 | 0.074 | 0.290 | 0.037 | 5,638 |

Table 4: Coefficients of variation (SD / mean) for 44,938 cervical vertebrae.

| Vertebra | VBHR | EPWR | FBDR | HWR | EPA | PSA | N |
| --- | --- | --- | --- | --- | --- | --- | --- |
| C2 | 0.037 | 0.059 | 0.034 | 0.063 | 0.443 | 0.029 | 7,705 |
| C3 | 0.049 | 0.036 | 0.036 | 0.085 | -0.884 | 0.032 | 8,180 |
| C4 | 0.047 | 0.034 | 0.033 | 0.081 | -0.682 | 0.03 | 8,017 |
| C5 | 0.046 | 0.036 | 0.035 | 0.086 | -0.482 | 0.03 | 8,127 |
| C6 | 0.046 | 0.032 | 0.032 | 0.091 | -0.817 | 0.027 | 7,695 |
| C7 | 0.035 | 0.03 | 0.027 | 0.074 | -16.01 | 0.021 | 5,214 |

Table 5: Results of multivariate analysis of variance (trimmed data) to determine the importance of multiple independent variables for explaining variability in the lumbar vertebral morphology metrics. The “F” columns provide the “F” statistic. The “P>F” columns provide the statistical significance. The F values document how much of the variability in the dependent variable is explained by each independent variable.

| Variable | VBHR R^2^ = 0.85 | | EPWR R^2^ = 0.92 | | FBDR R^2^ = 0.93 | | HWR R^2^ = 0.84 | | EPA R^2^ = 0.82 | | PSA R^2^ = 0.75 | |
| --- | --- | --- | --- | --- | --- | --- | --- | --- | --- | --- | --- | --- |
|  | F | P > F | F | P > F | F | P > F | F | P > F | F | P > F | F | P > F |
| *Vertebra* | 39009 | **0.000** | 85907 | **0.000** | 94108 | **0.000** | 34955 | **0.000** | 32422 | **0.000** | 20937 | **0.000** |
| Age | 54 | **0.000** | 133 | **0.000** | 72 | **0.000** | 1414 | **0.000** | 8 | **0.004** | 10 | **0.002** |
| Sex | 350 | **0.000** | 82 | **0.000** | 4 | **0.054** | 3969 | **0.000** | 535 | **0.000** | 146 | **0.000** |
| Race | 7 | **0.001** | 0 | 0.716 | 2 | 0.218 | 6 | **0.002** | 8 | **0.000** | 4 | **0.021** |
| Nation of Origin | 5 | **0.000** | 1 | 0.398 | 2 | **0.034** | 13 | **0.000** | 6 | **0.000** | 4 | **0.000** |
| Weight | 0 | 0.995 | 3 | 0.089 | 0 | 0.857 | 10 | **0.002** | 0 | 0.760 | 0 | 0.506 |
| Height | 0 | 0.829 | 7 | **0.010** | 2 | 0.211 | 2 | 0.194 | 1 | 0.458 | 0 | 0.526 |
| BMI | 0 | 0.645 | 3 | 0.076 | 0 | 0.868 | 0 | 0.482 | 0 | 0.911 | 0 | 0.496 |

Table 6: Results of multivariate analysis of variance to determine the importance of multiple independent variables for explaining variability in the cervical vertebral morphology metrics.

| Variable | VBHR R^2^=0.72 | | EPWR R^2^=0.77 | | FBDR R^2^=0.68 | | HWR R^2^=0.88 | | EPA R^2^=0.72 | | PSA R^2^=0.55 | |
| --- | --- | --- | --- | --- | --- | --- | --- | --- | --- | --- | --- | --- |
|  | F | P > F | F | P > F | F | P > F | F | P > F | F | P > F | F | P > F |
| Vertebra | 22659 | **0.000** | 30247 | **0.000** | 19163 | **0.000** | 66488 | **0.000** | 22852 | **0.000** | 10977 | **0.000** |
| Age | 136 | **0.000** | 161 | **0.000** | 421 | **0.000** | 2683 | **0.000** | 103 | **0.000** | 415 | **0.000** |
| Sex | 0 | 0.553 | 14 | **0.000** | 381 | **0.000** | 829 | **0.000** | 3 | 0.065 | 236 | **0.000** |
| Race | 7 | **0.001** | 0 | 0.928 | 0 | 0.895 | 27 | **0.000** | 7 | **0.001** | 1 | 0.472 |
| Nation of Origin | 2 | **0.017** | 1 | 0.223 | 7 | **0.000** | 21 | **0.000** | 2 | **0.039** | 6 | **0.000** |
| Weight | 18 | **0.000** | 5 | **0.029** | 0 | 0.958 | 4 | **0.044** | 24 | **0.000** | 1 | 0.303 |
| Height | 27 | **0.000** | 6 | **0.017** | 25 | **0.000** | 51 | **0.000** | 34 | **0.000** | 32 | **0.000** |
| BMI | 14 | **0.000** | 5 | **0.033** | 0 | 0.752 | 0 | 0.528 | 18 | **0.000** | 0 | 0.606 |

Table 7: Pearson’s correlation coefficients between morphologic variables for 35,275 lumbar vertebrae, after excluding vertebrae where any of the morphologic variables was ±2 SD from the average for all 42,980 vertebrae.

|  | VBHR | EPWR | FBDR | HWR | EPA | PSA |
| --- | --- | --- | --- | --- | --- | --- |
| VBHR | 1 |  |  |  |  |  |
| EPWR | 0.7741 | 1 |  |  |  |  |
| FBDR | 0.6694 | 0.9373 | 1 |  |  |  |
| HWR | 0.6661 | 0.8844 | 0.8712 | 1 |  |  |
| EPA | 0.9904 | 0.7338 | 0.6205 | 0.6551 | 1 |  |
| PSA | 0.7394 | 0.7911 | 0.9069 | 0.8069 | 0.7188 | 1 |

Table 8: Pearson’s correlation coefficients between morphologic variables for 44,938 cervical vertebrae, after excluding vertebrae where any of the morphologic variables was ±2 SD from the average for all 54,093 vertebrae.

|  | VBHR | EPWR | FBDR | HWR | EPA | PSA |
| --- | --- | --- | --- | --- | --- | --- |
| VBHR | 1 |  |  |  |  |  |
| EPWR | -0.6536 | 1 |  |  |  |  |
| FBDR | -0.2641 | 0.5298 | 1 |  |  |  |
| HWR | 0.7861 | -0.7548 | -0.4464 | 1 |  |  |
| EPA | 0.9848 | -0.5813 | -0.1828 | 0.7646 | 1 |  |
| PSA | 0.5704 | -0.352 | 0.5461 | 0.3203 | 0.6047 | 1 |

Table 9: The means and [standard deviations] for the absolute differences between adjacent lumbar vertebrae for each of the vertebral morphology metrics. These data include only those vertebrae with no abnormalities in any morphology metric using the data in Table 1. The sample size is smaller, since if either vertebra had any abnormality, that comparison between levels was excluded.

| Compare | N | VBHR | EPWR | FBDR | HWR | EPA | PSA |
| --- | --- | --- | --- | --- | --- | --- | --- |
| L1 to L2 | 3540 | 0.041[0.030] | 0.017[0.013] | 0.024[0.016] | 0.023[0.018] | 2.009[1.442] | 1.127[0.864] |
| L2 to L3 | 3635 | 0.034[0.026] | 0.018[0.014] | 0.018[0.013] | 0.023[0.019] | 1.611[1.224] | 1.136[0.847] |
| L3 to L4 | 3628 | 0.043[0.031] | 0.018[0.013] | 0.025[0.015] | 0.028[0.021] | 1.955[1.410] | 1.106[0.838] |
| L4 to L5 | 3554 | 0.124[0.059] | 0.026[0.019] | 0.020[0.015] | 0.027[0.022] | 5.191[2.443] | 1.583[1.178] |
| L5 to S1 | 3137 | 0.145[0.080] | 0.589[0.134] | 0.342[0.061] | 0.372[0.074] | 5.733[3.542] | 9.113[3.504] |

Table 10: The means and [standard deviations] for the absolute differences between adjacent cervical vertebrae for each of the vertebral morphology metrics. These data include only those vertebrae with no abnormalities in any morphology metric using the data in Table 2.

| Compare | N | VBHR | EPWR | FBDR | HWR | EPA | PSA |
| --- | --- | --- | --- | --- | --- | --- | --- |
| C2 to C3 | 4636 | 0.167[0.053] | 0.145[0.053] | 0.025[0.018] | 0.426[0.070] | 9.297[3.241] | 6.736[2.868] |
| C3 to C4 | 5039 | 0.041[0.031] | 0.032[0.024] | 0.027[0.019] | 0.052[0.038] | 2.063[1.553] | 1.983[1.475] |
| C4 to C5 | 5023 | 0.041[0.031] | 0.033[0.025] | 0.028[0.020] | 0.047[0.034] | 2.046[1.509] | 2.207[1.607] |
| C5 to C6 | 4850 | 0.045[0.033] | 0.036[0.026] | 0.034[0.022] | 0.044[0.034] | 2.429[1.732] | 2.466[1.719] |
| C6C7 | 3231 | 0.051[0.035] | 0.060[0.036] | 0.045[0.025] | 0.064[0.040] | 2.713[1.768] | 2.363[1.619] |

Figure 2: Box and whisker plots for the six morphology metrics calculated for 42,980 lumbar vertebrae. These plots document the types and magnitude of outliers in the data.


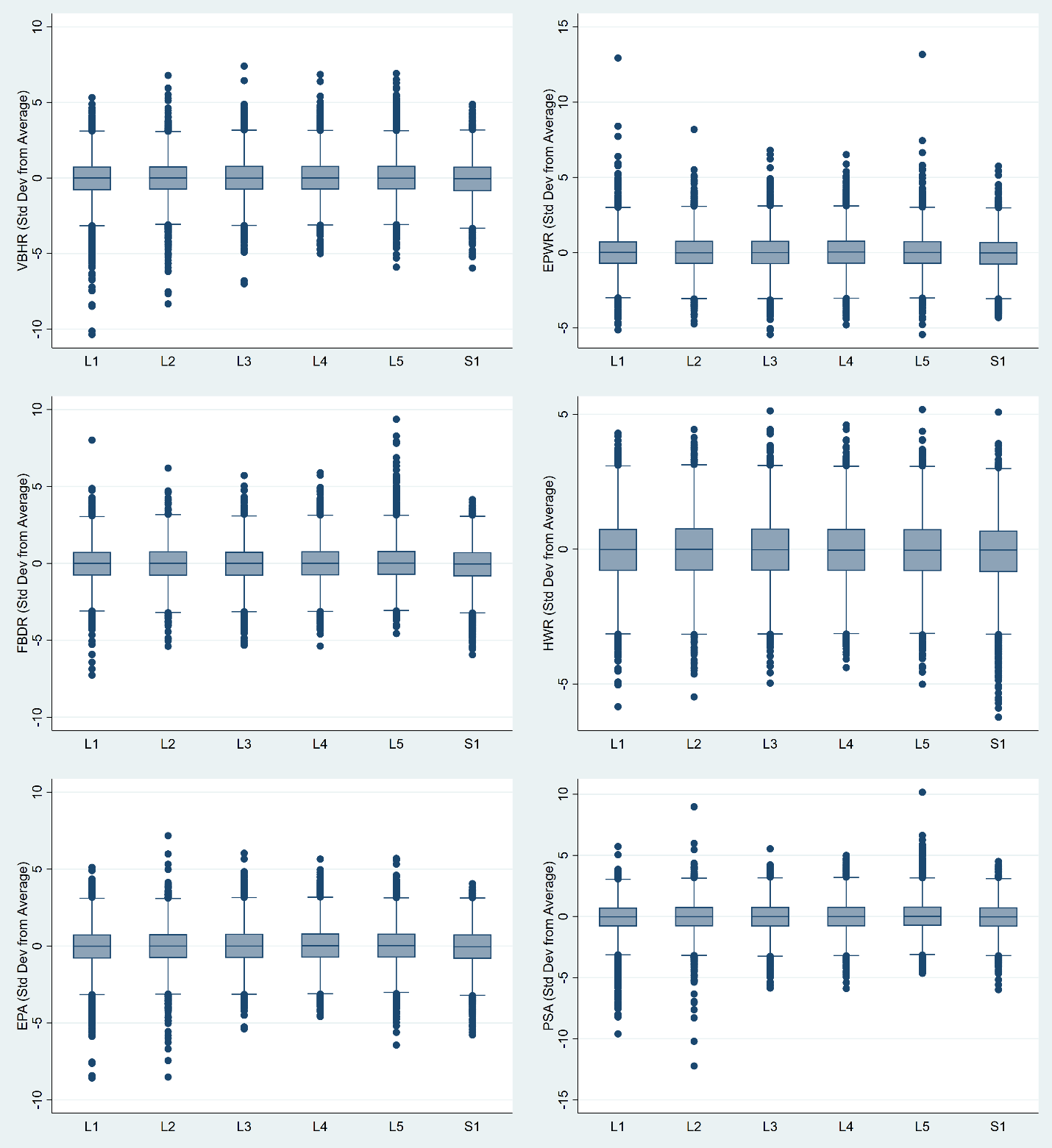


Figure 3: Box and whisker plots for the six morphology metrics calculated for 54,093 cervical vertebrae. These plots document the types and magnitude of outliers in the data.


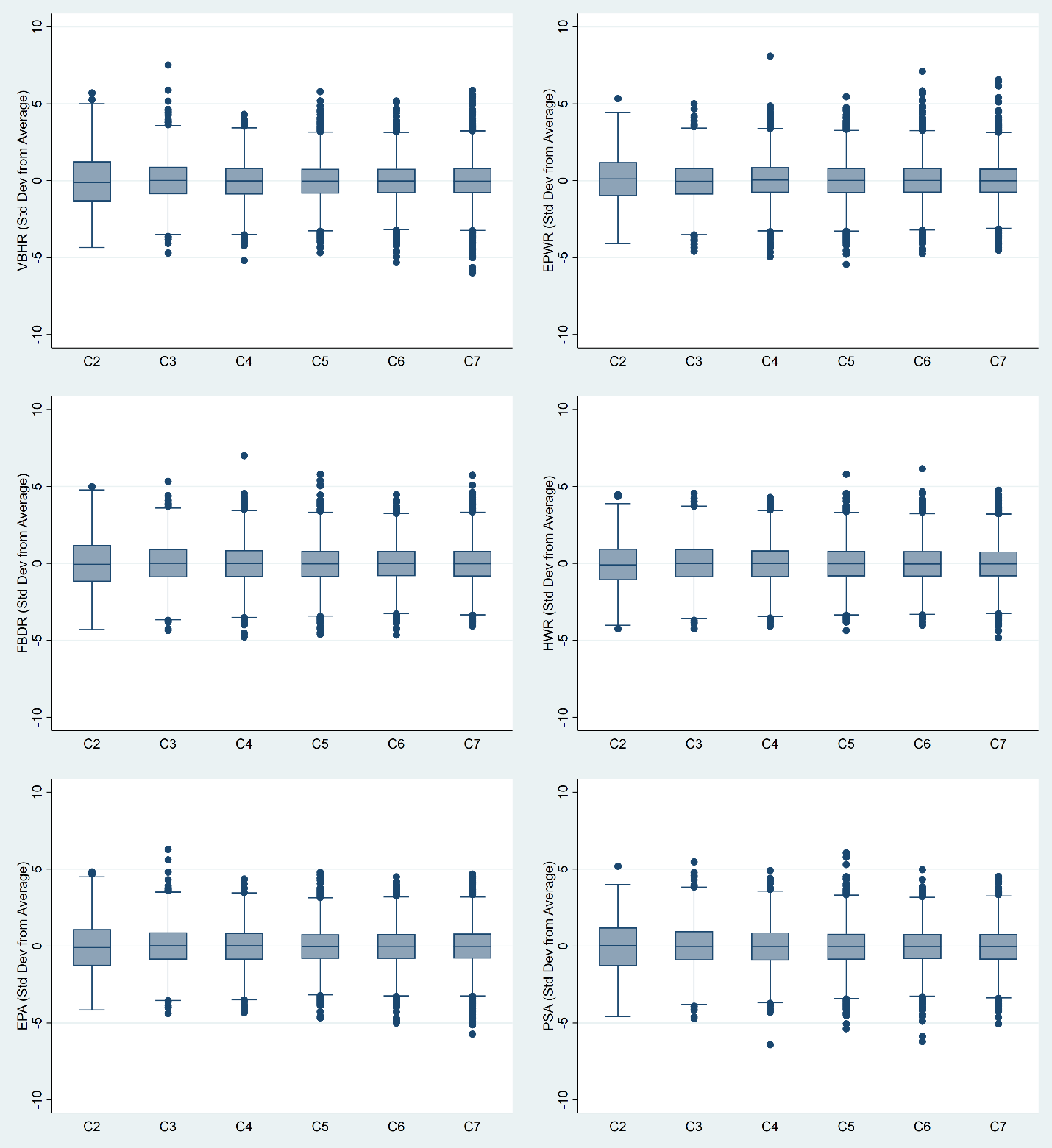


Table 11: Percent of all lumbar vertebrae in the NHANES-II study where the morphology metric was either > 2 or < -2 SD from the average, using data from Table 1 for the average and SD.

| Vert | Freq | VBHR | | EPWR | | FBDR | | HWR | | EPA | | PSA | |
| --- | --- | --- | --- | --- | --- | --- | --- | --- | --- | --- | --- | --- | --- |
|  |  | < -2 | > 2 | < -2 | > 2 | < -2 | > 2 | < -2 | > 2 | < -2 | > 2 | < -2 | > 2 |
| L1 | 7,037 | 7.1 | 3.3 | 3.9 | 4.9 | 4.8 | 4.0 | 4.9 | 4.0 | 6.5 | 3.5 | 6.8 | 3.6 |
| L2 | 7,324 | 5.5 | 4.8 | 3.9 | 5.0 | 4.9 | 4.2 | 4.6 | 4.3 | 5.1 | 4.9 | 5.5 | 4.3 |
| L3 | 7,318 | 4.5 | 5.8 | 3.9 | 5.1 | 5.2 | 4.2 | 4.5 | 4.3 | 4.2 | 5.6 | 5.4 | 4.8 |
| L4 | 7,333 | 3.6 | 5.8 | 3.8 | 5.4 | 4.5 | 4.4 | 4.7 | 4.0 | 3.9 | 5.4 | 5.0 | 5.0 |
| L5 | 7,181 | 3.9 | 5.9 | 3.5 | 5.4 | 3.2 | 6.1 | 4.8 | 4.1 | 4.4 | 5.4 | 4.4 | 6.4 |
| S1 | 6,787 | 6.1 | 4.6 | 8.7 | 3.8 | 8.7 | 3.8 | 8.1 | 3.0 | 5.5 | 3.9 | 5.5 | 4.0 |
| All | 42,980 | 5.1 | 5.0 | 4.6 | 4.9 | 5.2 | 4.5 | 5.2 | 4.0 | 4.9 | 4.8 | 5.4 | 4.7 |

Table 12: Percent of all cervical vertebrae in the NHANES-II study where the morphology metric was either > 2 or < -2 SD from the average, using data from Table 2 for the average and SD.

| Vert | Freq | VBHR | | EPWR | | FBDR | | HWR | | EPA | | PSA | |
| --- | --- | --- | --- | --- | --- | --- | --- | --- | --- | --- | --- | --- | --- |
|  |  | < -2 | > 2 | < -2 | > 2 | < -2 | > 2 | <-2 | >2 | < -2 | > 2 | < -2 | > 2 |
| C2 | 9,647 | 4.9 | 5.3 | 3.8 | 5.1 | 4.3 | 5.3 | 4.4 | 4.1 | 4.9 | 4.8 | 5.3 | 4.3 |
| C3 | 9,648 | 3.8 | 4.5 | 4.9 | 3.7 | 3.8 | 4.2 | 4.0 | 4.2 | 3.7 | 4.1 | 3.7 | 4.5 |
| C4 | 9,654 | 4.6 | 4.3 | 4.2 | 4.5 | 3.9 | 4.5 | 4.4 | 3.9 | 4.6 | 3.9 | 4.7 | 4.2 |
| C5 | 9,613 | 4.2 | 3.8 | 4.4 | 4.5 | 4.3 | 3.8 | 4.1 | 3.8 | 4.1 | 3.7 | 4.8 | 3.4 |
| C6 | 9,148 | 4.5 | 4.0 | 3.7 | 4.9 | 4.1 | 4.2 | 4.4 | 4.0 | 4.2 | 3.9 | 4.6 | 3.3 |
| C7 | 6,383 | 5.0 | 4.6 | 3.9 | 4.9 | 4.6 | 4.1 | 4.6 | 4.3 | 4.8 | 4.3 | 5.7 | 4.4 |
| All | 54,093 | 4.5 | 4.4 | 4.1 | 4.6 | 4.1 | 4.4 | 4.3 | 4.0 | 4.4 | 4.1 | 4.8 | 4.0 |
