# Supplementary material for: Definition of Normal Vertebral Morphometry Using NHANES‐II Radiographs
Source: JBMR Plus. 2022 Sep 27;6(10):e10677. doi: 10.1002/jbm4.10677 (PMC9549721; doi:10.1002/jbm4.10677)
Supplement: Supplementary file 2 — Appendix S2. Supporting information. [file JBM4-6-e10677-s003.docx]

# Supplemental Appendix 2: Reproducibility of vertebral morphology

To assess reproducibility of the metrics, the six morphology metrics were also calculated for flexion and extension radiographs for 380 asymptomatic volunteers. This is an expanded version of previously published data, with additional volunteers added using the same inclusion/exclusion criteria.[1] The morphology metrics were calculated from landmark coordinates obtained using the same neural networks and coded logic used with the NHANES-II X-rays. The morphology of each vertebra should not have changed between flexion and extension, unless there was an unhealed vertebral fracture, which is unlikely in an asymptomatic person. However, the position of each vertebra relative to the central beam path did change substantially between flexion and extension in many cases. The morphology measured from the flexion X-ray was compared to the morphology calculated from the extension X-ray using Bland-Altman limits of agreement. Any variability in the metrics is assumed to be error due to variability in radiographic projection.

The six morphology metrics calculated from flexion X-rays were compared to the metrics calculated from paired extension X-rays for 2,132 vertebrae from L1 to S1. Table 1 provides the results of the Bland-Altman limits of agreement analysis. This variability is assumed due to the differences in radiographic projection between images. Typically, there would not be much difference in radiographic projection between carefully obtained flexion versus extension X-rays, and this is reflected in the mean differences Table 1, but substantial differences did occur in some subjects, and this is reflected in the low and high limits of agreement. This experiment documents that some morphology metrics could vary by as much as 1.65 SD due to radiographic projection differences. Thus, it is important to strive for radiographs where the vertebrae of interest are positioned near the center of the radiograph. If changes in morphology are of interest, the radiographic projection should be similar between x-rays.

Table 1: Results of a Bland-Altman limits of agreement analysis for morphology (expressed as SD from the average) measured from 2,132 lumbar flexion X-rays compared to paired extension X-rays. The mean differences between the two measurements, as well as the low and high limits of agreement are provided. These data are specific to the methods used to obtain anatomic landmarks from the radiographs.

| Metric | Mean  Difference | Low | High |
| --- | --- | --- | --- |
| VBHR | 0.15 | -0.93 | 1.24 |
| EPWR | 0.028 | -1.59 | 1.65 |
| FBDR | -0.09 | -1.58 | 1.4 |
| HWR | -0.016 | -0.92 | 0.89 |
| EPA | 0.16 | -0.99 | 1.3 |
| PSA | 0.02 | -1.57 | 1.61 |

[1] Staub BN, Holman PJ, Reitman CA, Hipp JA. Sagittal plane lumbar intervertebral motion during seated flexion-extension in 658 asymptomatic, non-degenerated levels. J Neurosurgery Spine. 2015;23:731-8. doi:10.3171/2015.3.SPINE14898
