# Supplementary material for: Definition of Normal Vertebral Morphometry Using NHANES‐II Radiographs
Source: JBMR Plus. 2022 Sep 27;6(10):e10677. doi: 10.1002/jbm4.10677 (PMC9549721; doi:10.1002/jbm4.10677)
Supplement: Supplementary file 3 — Appendix S3. Supporting information. [file JBM4-6-e10677-s001.docx]

# Supplemental Appendix 3: Errors due to variability in radiographic projection

To further document expected variability in the metrics specifically due to variations in radiographic projections that can occur in clinical practice, digitally reconstructed radiographs (DRR) were used. Thin-slice (1 mm or less) anonymized computed tomography exams of the lumbar spine of 26 individuals or cadavers (from past research studies) were interpolated to 0.2 mm isotropic resolution. The three-dimensional coordinates of the four anatomic landmarks described in Figure 1 were digitized, in 3D, for the mid-sagittal plane of each vertebra from L1 to S1, using intersecting axial, coronal, and sagittal slices. Landmarks were thus placed in the mid-sagittal plane at clear and well-defined locations. This may be as close as possible to “gold standard” landmark placement. The image processing and landmark digitization was completed using Slicer 3D.[59] Custom python code was developed to create 2D, digitally reconstructed radiographs (DRR) from the 3D data using program calls to Plastimatch DRR[60]. The projection matrices used by Plastimatch DRR to create the X-rays were also used to calculate, from the known 3D coordinates, the precise coordinates of each landmark on each 2D simulated X-ray. A 40 inch source-to-image distance was used for all X-rays. The base x-ray was centered on the centroid of the L3 vertebra (this is the baseline isocenter). Forty additional X-rays were generated with the following variations:

- random beam tilts of between ± 10 deg in each of the sagittal and axial planes
- random displacements from the baseline isocenter ± 2 endplate widths (EPW) in the AP and LR directions, plus ± 3 EPW shifts in the cranial-caudal direction
- random image rotations between ± 24 deg

These variations resulted in a wide range of radiographic projections. The landmarks in every image were precisely known so there were zero errors in 2D landmark coordinates relative to the 3D coordinates. This allows for documenting the effect of variability in vertebral morphology metrics that are due to radiographic projection, without uncertainty in landmark placement. These precisely calculated landmarks were analyzed to obtain the six vertebral body morphology metrics. There were thus 41 morphology measurements for every vertebra from L1 to S1 for each of 26 independent CT exams. The variability in each metric was assessed using the standardized version of the metric, since all of these metrics are thereby expressed as standard deviations from the average and that allows for combining data for all vertebrae and for consistent interpretation of the data. Although the coordinates of landmarks in every simulated X-ray were precisely calculated, the quality of the simulated radiographs was below clinical standards and not appropriate for use toward validating the accuracy of neural networks in obtaining landmarks.

Figure 1: Details of anatomic landmark placement. Dashed lines show the assumed mid-sagittal plane of the superior and inferior endplates, identified as bisecting the radiographic shadows of the left and right sides of the endplates (yellow arrows). The red circles show the four landmarks used to measure vertebral body morphology. The red arrow points to an anterior osteophyte that is ignored. The dotted lines show the anterior and posterior vertebral body heights.


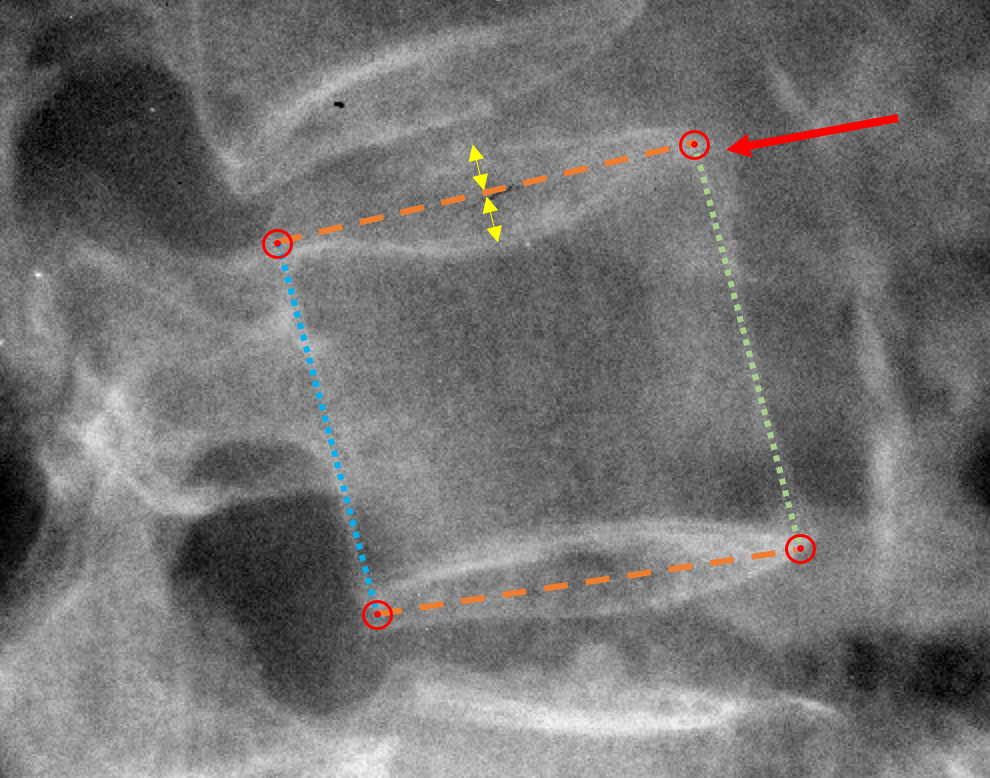


## Results and Discussion

The experiment using simulated X-rays was completed to help understand how variability in radiographic projection can affect the vertebral morphology metrics. This could be important, for example, with sub-optimally positioned patients, or in patients with scoliosis or other deformities, all of which can result in the x-ray beam passing from source to image obliquely to the vertebral endplates or the posterior wall of the vertebrae. Conventional measures of variability, such as the coefficient of variation (CV = SD / mean) or the coefficient of quartile variation (CQV = 100* (Q_3_ – Q_1_)/ (Q_3_ + Q_1_)) were not used as the mean values and (Q_3_ + Q_1_) were close to zero for some of the standardized metrics and CV or QCV are uninterpretable when the denominator approaches zero. Instead, the median and the range (max – min) for the standardized metrics was calculated for the 41 simulated X-rays that were generated from each CT exam. The median and 95^th^ percentile for these ranges, across the 26 CT exams, are provided in Table 1. For example, due to a wide range in radiographic projections, EPA will typically vary by 0.37 SDs but could vary by as much as 1.1 SDs from the average EPA in a normal vertebra. The observed ranges of standardized metrics due to variability in radiographic projection was largest for FBDR and PSA. The high median values for FBDR and PSA are likely due to the very low variability in these metrics between individuals in the NHANES-II study and the much higher variability in radiographic projection in the simulated x-rays compared to the NHANES-II x-rays that were all obtained following a strict protocol. It is thus relatively easy to get 2+ SD differences due to radiographic projection. This is not as much a weakness in the metrics as it is evidence of the need for good quality radiographs and the need to use caution when interpreting these metrics in the presence of substantial out-of-plane imaging. It is of course possible that strategies can be developed for mitigating the effect of variability in radiographic projection. One option would be a neural network that determines the type and magnitude of out-of-plane and uses that data to correct the morphology metrics.

Table 1: Variability in standardized metrics (SD from Average Normal) due to variability in radiographic projection.

| Metric | Median | 95^th^ Percentile |
| --- | --- | --- |
| EPA | 0.3658 | 1.0796 |
| EPWR | 0.6643 | 2.2362 |
| FBDR | 2.4421 | 4.1485 |
| HWR | 0.6378 | 1.1342 |
| PSA | 2.1198 | 3.7636 |
| VBHR | 0.3628 | 1.2465 |
